# Persistence of Resistant *Staphylococcus epidermidis* after Single Course of Clarithromycin

**DOI:** 10.3201/eid1109.050124

**Published:** 2005-09

**Authors:** Maria Sjölund, Eva Tano, Martin J. Blaser, Dan I. Andersson, Lars Engstrand

**Affiliations:** * University Hospital, Uppsala, Sweden;; † The Swedish Institute for Infectious Disease Control, Solna, Sweden;; ‡ New York University School of Medicine, New York, New York, USA

**Keywords:** antimicrobial resistance, Staphylococcus epidermidis, macrolides, clarithromycin, research

## Abstract

We examined how a common therapy that includes clarithromycin affects normally colonizing *Staphylococcus epidermidis*. Samples from the nostrils of 5 patients receiving therapy were collected before, immediately after, 1 year after, and 4 years after treatment. From each patient and sample, *S. epidermidis* strains were isolated and analyzed for clarithromycin susceptibility and presence of the *erm*(C) gene. We show that macrolide-resistant strains of *S. epidermidis* were selected during therapy and that the same resistant strain may persist for 4 years, in the absence of further antimicrobial treatment.

## Abstract 2

Short course of antimicrobial therapy can select resistant bacteria that persist for 4 years or longer.

The emergence and spread of drug-resistant bacteria pose a serious threat to global public health (*1**,**2*), and the normal biota constitutes a potential reservoir of resistance genes that can spread to invading pathogens (*3**,**4*). A gene (*aphA*-*3*) that confers resistance to amikacin and kanamycin in *Campylobacter* spp. may have originated from the gram-positive *Enterococcus*, *Streptococcus*, or *Staphylococcus* spp (*4*). Similarly, *aadE* and *tet*(*O*), which encode streptomycin and tetracycline resistance, respectively, have been found in *Campylobacter* spp. but are considered to have been transferred from gram-positive bacteria (*4*). Moreover, parts of the mosaic penicillin-binding protein genes of *Streptococcus pneumoniae* that confer penicillin resistance are likely to originate from viridans streptococci, which tend to be more resistant (*5*), and the *mecA* gene that renders *Staphylococcus aureus* resistant to all β-lactams likely originated in coagulase-negative staphylococci (*6*).

*Staphylococcus epidermidis*, a coagulase-negative staphylococcus, is a major component of the normal human biota (*7*). Large populations (10^3^–10^6^ CFU/cm^2^) of *S. epidermidis* are commonly found in the anterior nares and the axillae (*7*). Coagulase-negative staphylococci have been increasingly recognized as important nosocomial pathogens (*8*), affecting immunocompromised patients or those with indwelling devices, such as joint prostheses, prosthetic heart valves, and central venous catheters (*8**,**9*). Since the infections associated with *S. epidermidis* are chiefly acquired during hospitalization, it is not surprising that they are increasingly resistant to antimicrobial drugs (*10*). Macrolide resistance in *S. epidermidis* is commonly caused by *erm* genes (*10*), whose products dimethylate a 23S rRNA adenine residue, preventing macrolide binding to the 50S ribosomal subunit (*11**,**12*). In *S. epidermidis*, *erm*(C), which induces high-level macrolide resistance, predominates (*13**,**14*).

In this study, we have assessed how a commonly used therapy that includes clarithromycin affects the normal microbiota of *S. epidermidis*. We show that a 1-week course of clarithromycin selects for macrolide-resistant *S. epidermidis* that may persist up to 4 years after treatment.

## Methods

During a cohort study that examined eradication of *Helicobacter pylori* by a combination therapy that included clarithromycin, we chose 5 patients in order to study macrolide resistance in *S. epidermidis*. In the larger study, all patients were colonized with *H. pylori* and had either a duodenal or gastric ulcer, for which a 7-day course of clarithromycin 250 mg twice per day (b.i.d.), metronidazole 400 mg b.i.d., and omeprazole 20 mg b.i.d. was given. We excluded patients who had previously been treated for *H. pylori* or who had received any antimicrobial treatment within the prior 4 weeks. The control group included 5 patients with dyspeptic symptoms who had not received any antimicrobial treatment. During the 4-year course of this study, no other antimicrobial treatment was allowed. The study was approved by the human ethics committee at Uppsala University, Uppsala, Sweden.

Samples from the nares of each patient were collected 1 day before treatment, 3–7 days immediately after, 1 year later, and 4 years later. All samples were stored at –70°C until analyzed. From each study patient and each sample, 10 independent colonies of *S. epidermidis* were isolated on Columbia blood agar plates (Difco, Baltimore, MD, USA) and verified by Gram staining, positive catalase, negative DNase, negative mannitol, and negative trehalose testing. DNA was extracted from the bacterial strains with the DNeasy Tissue kit (Qiagen, Hilden, Germany). MIC of clarithromycin was measured with the Etest (AB Biodisk, Solna, Sweden), as recommended by the Swedish Reference Group for Antibiotics.

The *erm*(C) gene was detected as described (*13*), by using primers ermC1/C2 5´-GCTAATATTGTTTAAATCGTCAATTCC-3´ and 5´-GGATCAGGAAAAGGACATTT-3´ but with the following modifications: each polymerase chain reaction (PCR) contained 25 μL master mix (PCR Master, Roche, Penzberg, Germany), 30 pmol of each primer, 14 μL distilled water, and 5 μL DNA sample. The amplified 572-bp product was separated by electrophoresis on a 1.5% agarose gel.

For pulsed-fiedl gel electrophoresis (PFGE), bacterial cells were harvested by centrifugation from 3 mL overnight cultures in brain-heart infusion broth and resuspended in 3 mL Tris-HCl buffer (pH 7.6). The bacterial suspension (150 μL) was mixed with 150 μL 2% agarose (Sigma, St. Louis, MO, USA) in Tris-HCl buffer and used for making the gel plugs. The plugs were incubated at 35°C overnight in 4 mL Lysis 1 buffer (6 mmol/L Tris-HCl [pH 7.6], 1 mol/L NaCl, 100 mmol/L EDTA [pH 7.5], 0.5% Brij 58, 0.2% deoxycholate, 0.5% sodium lauryl sarcosine [Sarcosyl, Kodak International Biotechnologies, New Haven, CT, USA], 1 mg/mL lysozyme [Life Technology, Sigma-Aldrich, Steinheim, Germany], and 7 μg/mL lysostaphin [Sigma]), then incubated overnight at 55°C in 4 mL Lysis 2 buffer (1% sodium lauryl sarcosine [Sarcosyl], 0.5 mol/L EDTA [pH 9.5], and 50 μg/mL Proteinase K [Roche Diagnostics Corporation, Indianapolis, IN, USA]). The plugs were washed 3 times for >30 min at 35°C in 4 mL of Tris-EDTA buffer. A 3-mm slice of each gel plug was incubated overnight at 25°C with *Sma*I (Life Technology, Invitrogen, Carlsbad, CA, USA) and buffer, then placed in the wells of a 1.0% agarose gel (Ultra pure agarose, Life Technology, Invitrogen), sealed with 1.0 % agarose, and put in 0.5× Tris-borate-EDTA buffer. Electrophoresis (Gene Path Electrophoresis System, Bio-Rad Laboratories, Inc., Hercules, CA, USA) was performed with the following conditions: 5–60 s switch interval with a voltage gradient of 6 V/h at an angle of 120° for 23 h. After electrophoresis, the gel was stained with ethidium bromide for 30 min, destained in distilled water for 1 h, and DNA was visualized under UV light (Gel doc 1000, Bio-Rad Laboratories, Inc.). The restriction fragment profiles were interpreted by comparison with each other, with a reference *S. aureus* strain NCTC 8325, and with a λ phage DNA standard (New England Biolabs, Beverly, MA, USA).

## Results

At 1 day before treatment, all 5 patients in the treatment group harbored clarithromycin-susceptible (MIC <0.5 μg/mL) *S. epidermidis* among the 10 independent colonies examined. In 4 patients, all 10 isolates were susceptible, but in the fifth patient 2 isolates were highly resistant (MIC >256 μg/mL) because *erm*(C) was present. Immediately after completing treatment, 4 of 5 patients displayed high-level clarithromycin-resistant (MIC >256 μg/mL) isolates (Table 1). The other isolates from this time point were either resistant with lower MIC values (16–96 μg/mL) or susceptible. Highly resistant isolates could be detected 1 year after treatment in 4 patients and 4 years after treatment in 3 patients. All highly resistant isolates harbored *erm*(C), as determined by PCR. In the controls, who did not receive any antimicrobial treatment, no selection of resistant staphylococci was detected. However, in control 4, 1 highly resistant isolate was detected at the first time point. In the same control, 2 of 10 isolates were highly resistant at the second time point, but no resistance was detected at the 1- and 4-year follow-ups. In control patient 5, all isolates were susceptible, except 5 resistant isolates detected at the 4-year follow up (Table 2).

**Table 1:** Characteristics of *Staphylococcus epidermidis* isolated from patients*†

Patient	Pretreatment‡			Posttreatment‡			1 year posttreatment‡			4 years posttreatment‡		
	Isolate	MIC	DNA	Isolate	MIC	DNA	Isolate	MIC	DNA	Isolate	MIC	DNA
1	1A:1	0.094	A	1B:1	0.094	F	1C:1	>256	H	1D:1	>256	H
	1A:2	0.094	B	1B:2	0.094	F	1C:2	>256	H	1D:2	0.094	M
	1A:3	0.125	B	1B:3	0.125	G	1C:3	32	K	1D:3	0.094	M
	1A:4	0.125	B	1B:4	0.094	F	1C:4	>256	H	1D:4	0.094	B
	1A:5	0.125	C	1B:5	>256	H	1C:5	>256	H	1D:5	>256	H
	1A:6	0.094	B	1B:6	0.094	J	1C:6	>256	H	1D:6	0.094	C
	1A:7	0.094	D	1B:7	0.094	F	1C:7	>256	H	1D:7	0.094	M
	1A:8	0.094	D	1B:8	0.125	B	1C:8	>256	L	1D:8	0.094	G
	1A:9	0.125	B	1B:9	>256	H	1C:9	>256	H	1D:9	>256	H
	1A:10	0.064	E	1B:10	0.094	G	1C:10	>256	H	1D:10	>256	H
2	2A:1	>256	N	2B:1	>256	S	2C:1	>256	N	2D:1	0.094	P
	2A:2	0.125	O	2B:2	>256	N	2C:2	>256	N	2D:2	0.094	T
	2A:3	0.125	O	2B:3	>256	N	2C:3	>256	N	2D:3	>256	N
	2A:4	>256	N	2B:4	>256	S	2C:4	>256	N	2D:4	>256	N
	2A:5	0.125	O	2B:5	>256	S	2C:5	>256	N	2D:5	0.125	O
	2A:6	0.094	P	2B:6	>256	N	2C:6	0.094	T	2D:6	0.125	P
	2A:7	0.19	Q	2B:7	96	S	2C:7	0.125	T	2D:7	0.094	T
	2A:8	0.125	R	2B:8	0.094	T	2C:8	>256	N	2D:8	0.125	P
	2A:9	0.094	P	2B:9	>256	N	2C:9	>256	N	2D:9	>256	N
	2A:10	0.094	P	2B:10	>256	S	2C:10	>256	N	2D:10	0.094	P
3	3A:1	0.125		3B:1	32		3C:1	0.125		3D:1	0.125	
	3A:2	0.125		3B:2	32		3C:2	>256		3D:2	>256	
	3A:3	0.125		3B:3	24		3C:3	0.25		3D:3	0.25	
	3A:4	0.094		3B:4	16		3C:4	>256		3D:4	>256	
	3A:5	0.125		3B:5	16		3C:5	>256		3D:5	>256	
	3A:6	0.125		3B:6	24		3C:6	0.094		3D:6	0.094	
	3A:7	0.125		3B:7	24		3C:7	>256		3D:7	>256	
	3A:8	0.125		3B:8	24		3C:8	>256		3D:8	>256	
	3A:9	0.125		3B:9	24		3C:9	0.125		3D:9	0.125	
	3A:10	0.19		3B:10	16		3C:10	0.125		3D:10	0.125	
4	4A:1	0.125		4B:1	24		4C:1	0.125		4D:1	0.125	
	4A:2	0.094		4B:2	>256		4C:2	0.125		4D:2	0.125	
	4A:3	0.125		4B:3	24		4C:3	0.125		4D:3	0.125	
	4A:4	0.125		4B:4	>256		4C:4	0.125		4D:4	0.125	
	4A:5	0.125		4B:5	24		4C:5	0.125		4D:5	0.125	
	4A:6	0.125		4B:6	>256		4C:6	0.125		4D:6	0.125	
	4A:7	0.125		4B:7	24		4C:7	0.094		4D:7	0.094	
	4A:8	0.094		4B:8	>256		4C:8	0.094		4D:8	0.125	
	4A:9	0.094		4B:9	24		4C:9	0.125		4D:9	0.125	
	4A:10	0.125		4B:10	24		4C:10	0.125		4D:10	0.094	
5	5A:1	0.125		5B:1	>256		5C:1	>256		5D:1	0.19	
	5A:2	0.125		5B:2	>256		5C:2	>256		5D:2	0.125	
	5A:3	0.125		5B:3	>256		5C:3	>256		5D:3	0.064	
	5A:4	0.125		5B:4	>256		5C:4	>256		5D:4	0.094	
	5A:5	0.125		5B:5	>256		5C:5	>256		5D:5	0.125	
	5A:6	0.125		5B:6	>256		5C:6	0.094		5D:6	0.125	
	5A:7	0.094		5B:7	>256		5C:7	0.125		5D:7	0.125	
	5A:8	0.125		5B:8	>256		5C:8	>256		5D:8	0.094	
	5A:9	0.125		5B:9	>256		5C:9	>256		5D:9	0.094	
	5A:10	0.094		5B:10	>256		5C:10	>256		5D:10	0.094	

*Isolates from only 2 patients were DNA fingerprinted. †For each patient and time point, 10 independent isolations of *S. epidermidis* were performed. ‡Clarithromycin MIC measured by Etest. DNA fingerprinting by pulsed-field gel electrophoresis; different patterns are indicated as letters.

**Table 2:** Characteristics of *Staphylococcus epidermidis* isolated from controls*†

Control	Pretreatment‡			Posttreatment‡			1 year posttreatment‡			4 years posttreatment‡		
	Isolate	MIC	DNA	Isolate	MIC	DNA	Isolate	MIC	DNA	Isolate	MIC	DNA
1	1A:1	0.064	–	1B:1	0.064	–	1C:1	0.094	FF	1D:1	0.064	JJ
	1A:2	0.064	AA	1B:2	0.064	BB	1C:2	0.094	GG	1D:2	0.047	JJ
	1A:3	0.064	–	1B:3	0.094	–	1C:3	0.047	GG	1D:3	0.064	JJ
	1A:4	0.064	BB	1B:4	0.064	FF	1C:4	0.064	HH	1D:4	0.064	JJ
	1A:5	0.064	CC	1B:5	0.047	BB	1C:5	0.064	HH	1D:5	0.094	JJ
	1A:6	0.064	DD	1B:6	0.047	FF	1C:6	0.032	HH	1D:6	0.064	JJ
	1A:7	0.047	–	1B:7	0.064	–	1C:7	0.032	HH	1D:7	0.064	JJ
	1A:8	0.064	–	1B:8	0.064	FF	1C:8	0.047	HH	1D:8	0.064	JJ
	1A:9	0.047	EE	1B:9	0.064	FF	1C:9	0.047	HH	1D:9	0.064	JJ
	1A:10	0.064	CC	1B:10	0.047	–	1C:10	0.047	HH	1D:10	0.064	JJ
2	2A:1	0.094	KK	2B:1	0.094	KK	2C:1	0.094	LL	2D:1	0.094	KK
	2A:2	0.047	LL	2B:2	0.064	LL	2C:2	0.047	OO	2D:2	0.094	KK
	2A:3	0.094	LL	2B:3	0.064	–	2C:3	0.047	OO	2D:3	0.094	LL
	2A:4	0.047	KK	2B:4	0.064	LL	2C:4	0.047	OO	2D:4	0.094	MM
	2A:5	0.064	KK	2B:5	0.064	LL	2C:5	0.064	LL	2D:5	0.094	LL
	2A:6	0.064	MM	2B:6	0.064	LL	2C:6	0.064	PP	2D:6	0.094	KK
	2A:7	0.064	KK	2B:7	0.064	LL	2C:7	0.047	PP	2D:7	0.094	LL
	2A:8	0.094	LL	2B:8	0.047	KK	2C:8	0.047	PP	2D:8	0.125	KK
	2A:9	0.064	KK	2B:9	0.064	NN	2C:9	0.125	LL	2D:9	0.064	KK
	2A:10	0.094	KK	2B:10	0.032	NN	2C:10	0.047	OO	2D:10	0.094	KK
3	3A:1	0.125		3B:1	0.125		3C:1	0.125		3D:1	0.125	
	3A:2	0.125		3B:2	0.125		3C:2	0.125		3D:2	0.125	
	3A:3	0.125		3B:3	0.125		3C:3	0.125		3D:3	0.125	
	3A:4	0.125		3B:4	0.125		3C:4	0.125		3D:4	0.125	
	3A:5	0.125		3B:5	0.125		3C:5	0.125		3D:5	0.25	
	3A:6	0.125		3B:6	0.125		3C:6	0.25		3D:6	0.25	
	3A:7	0.125		3B:7	0.25		3C:7	0.25		3D:7	0.25	
	3A:8	0.25		3B:8	0.25		3C:8	0.25		3D:8	0.25	
	3A:9	0.25		3B:9	0.25		3C:9	0.25		3D:9	0.25	
	3A:10	0.25		3B:10	0.25		3C:10	0.25		3D:10	0.25	
4	4A:1	0.125		4B:1	0.125		4C:1	0.125		4D:1	0.125	
	4A:2	0.125		4B:2	0.125		4C:2	0.125		4D:2	0.125	
	4A:3	0.125		4B:3	0.125		4C:3	0.125		4D:3	0.125	
	4A:4	0.125		4B:4	0.125		4C:4	0.125		4D:4	0.125	
	4A:5	0.25		4B:5	0.125		4C:5	0.125		4D:5	0.125	
	4A:6	0.25		4B:6	0.25		4C:6	0.125		4D:6	0.125	
	4A:7	0.25		4B:7	0.25		4C:7	0.125		4D:7	0.125	
	4A:8	0.25		4B:8	0.25		4C:8	0.25		4D:8	0.125	
	4A:9	0.25		4B:9	>256		4C:9	0.25		4D:9	0.125	
	4A:10	>256		4B:10	>256		4C:10	0.25		4D:10	0.125	
5	5A:1	0.125		5B:1	0.125		5C:1	0.125		5D:1	0.125	
	5A:2	0.125		5B:2	0.125		5C:2	0.125		5D:2	0.125	
	5A:3	0.125		5B:3	0.125		5C:3	0.125		5D:3	0.125	
	5A:4	0.125		5B:4	0.125		5C:4	0.125		5D:4	0.125	
	5A:5	0.125		5B:5	0.125		5C:5	0.125		5D:5	128	
	5A:6	0.125		5B:6	0.125		5C:6	0.125		5D:6	128	
	5A:7	0.125		5B:7	0.125		5C:7	0.125		5D:7	128	
	5A:8	0.125		5B:8	0.125		5C:8	0.125		5D:8	128	
	5A:9	0.125		5B:9	0.125		5C:9	0.125		5D:9	0.064	
	5A:10	0.25		5B:10	0.125		5C:10	0.125		5D:10	64	

*Isolates from only 2 controls were DNA fingerprinted. †Time points are defined with respect to treatment, although the control group received no treatment. For each control and time point, 10 independent isolations of *S. epidermidis* were performed. ‡Clarithromycin MIC measured by Etest. DNA fingerprinting by pulsed-field gel electrophoresis; different patterns are indicated as letters. –, not determined.

Isolates obtained from patients 1 and 2, chosen to investigate the clonality of resistance, were genotyped by pulsed-field gel electrophoresis (PFGE). Before treatment, each patient carried 5 different *S. epidermidis* strains among the 10 colonies tested. In patient 1, no highly resistant isolates were detected before treatment. However, immediately after treatment, 2 of 10 isolates were highly resistant, both defined as strain H. Based on PFGE patterns, strain H was detected in 8 of 10 isolates 1 year after treatment and in 4 of 10 isolates 4 years after treatment (Table 1). Strain G, which was susceptible to clarithromycin, was present immediately after treatment and 4 years later. Two of the pretreatment strains (B and C) were detected 4 years after treatment.

For patient 2, from whom 2 highly resistant isolates with the same profile (N) were detected before treatment, PFGE showed 2 distinct resistant strains (N and S) to be present immediately after treatment. Clone N was detected in 8 of 10 isolates 1 year after treatment and in 3 of 10 isolates 4 years after treatment. Susceptible strains P and Q, which were present pretreatment, were isolated again 4 years after treatment. Thus, after treatment in both cases, PFGE analysis showed that highly resistant strains persisted for 4 years, in the absence of further selection pressure, and that both resistant and susceptible strains were present 4 years after treatment (Table 1).

In a similar manner, the isolates from 2 controls were genotyped (Table 2). In control 1, at least 5 different strains were present at the start of the study. After 1 year, the composition had changed, and after 4 years, a new strain predominated in the flora. In control 2, we initially detected 3 different strains. These strains were also represented at each time point and predominated at 4 years. Thus, 1 control showed stable populations, whereas the other showed a dynamic state in the absence of treatment.

## Discussion

Since antimicrobial drugs do not distinguish between pathogenic and colonizing bacteria, our indigenous biota is affected every time a drug is given (*3**,**15*). Resistance development in staphylococci that normally colonize the skin has previously been observed after antimicrobial prophylaxis or treatment (*16**–**18*). Depending on mechanism, resistance can be selected de novo, exist in the pretreatment biota, or be acquired, especially in hospital environments.

In this study of the effect of a 1-week course of clarithromycin on indigenous *S. epidermidis* populations, we show that macrolide-resistant *S. epidermidis* strains are selected during therapy and that, without further selection, resistant clones can persist for up to 4 years. This finding is important for several reasons. First, although *S. epidermidis* belongs to the normal cutaneous microbiota, it may be a pathogen, especially in hospitalized patients (*8*); stably resistant populations increase the risk for treatment failure. Second, resistance in the normal microbiota might contribute to increased resistance in higher-grade pathogens by interspecies genetic transfer. Since the population size of the normal microbiota is large, multiple and different resistant variants can develop, which increases the risk for spread to populations of pathogens. Persisting populations of resistant microbiota further enhance transfer risk, especially if the selecting agent is used for treatment. Third, antimicrobial drugs may affect the stability of residential populations.

Whether a resistant population persists is mostly determined by the fitness and transmission costs of resistance (*19**,**20*). Most resistance involves a cost (*21**–**24*), but resistance may occur without detectable cost (*25*). If most resistance is costly for bacteria, resistant populations should decline once the selective antimicrobial pressure is removed. However, mutations may arise that compensate for the fitness cost, restoring the bacteria's fitness without reversion of the resistant phenotype. This phenomenon, compensatory evolution, is considered to be relevant to stabilizing resistant populations (*26*). Other important mechanisms that could stabilize resistant populations are no-cost resistance mutations (*25*) and genetic linkage with adjacent genes. Despite substantially decreased sulfonamide use in the United Kingdom from 1991 to 1999, *Escherichia coli* resistance to sulfonamides remained high (39.7% in 1991, 46.0% in 1999) because sulfonamide resistance was linked to other resistance genes that continued to be under selective pressure (*27*). In poultry, since *vanA* can be co-selected with *erm*(B) in *Enterococcus hirae* isolates (*28*), vancomycin resistance can be maintained by using macrolides, despite excluding avoparcin from animal feed. Thus, the stability and maintenance of antimicrobial drug resistance depends on the magnitude of selective pressure, compensatory evolution, no-cost associated resistance, and genetic linkage with co-selected resistance genes.

In our study, resistant isolates persisted long after drug treatment was completed. However, a variation in length of persistence between the patients was observed. Whether this variation is related to different costs associated with *erm*(C) carriage or different extents of genetic compensation for an initial cost cannot be concluded from current data. The observed variation in persistence of resistance could further be affected by the degree of recolonization and transient colonization of new strains during the 4-year study period. Although recolonization with *S. epidermidis* is presumably low, it can be enhanced by, for example, nosocomial spread during hospital stays (*29*). That indigenous *S. epidermidis* populations may naturally change in composition over time was reflected in the control group. According to the PFGE profiles from controls 1 and 2, populations of *S. epidermidis* can either remain stable for 4 years or show a more dynamic state, with new strains appearing over time. A change in the composition of the flora was also observed in control 5, in whom 5 resistant isolates appeared in the susceptible flora after 4 years. Since this patient did not receive any antimicrobial drugs during the study period, this finding is likely due to recolonization or transient colonization of a strain from the environment. However, most importantly, although a few resistant isolates were detected among the controls, no selection of resistant *S. epidermidis* occurred over the 4-year study period, as was observed in the treated patients.

In conclusion, antimicrobial drug treatment affects our indigenous microbiota and can give rise to long-term colonization with resistant populations. Our results show that as part of a combination therapy, a 7-day course of clarithromycin resulted in macrolide-resistant *S. epidermidis* that persisted up to 4 years without any further selection. In total, these observations suggest that selection of resistance in our microbiota after short antimicrobial drug courses may not be a rare phenomenon. However, the extent, to which other antimicrobial treatment regimens select for resistant *S. epidermidis* remains to be investigated.
